# Novel Silica-Based Hybrid Adsorbents: Lead(II) Adsorption Isotherms

**DOI:** 10.1155/2013/897159

**Published:** 2013-11-04

**Authors:** Junsheng Liu, Xin Wang

**Affiliations:** Key Laboratory of Membrane Materials & Processes, Department of Chemical and Materials Engineering, Hefei University, 99 Jinxiu Road, Hefei Economic and Technological Development Zone, Hefei 230601, China

## Abstract

Water pollution caused by the lead(II) from the spent liquor has caught much attention. The research from the theoretical model to application fundaments is of vital importance. In this study, lead(II) adsorption isotherms are investigated using a series of hybrid membranes containing mercapto groups (–SH groups) as the hybrid adsorbents. To determine the best fitting equation, the experimental data were analyzed using six two-parameter isotherm equations (i.e., Langmuir, Freundlich, Dubinin-Radushkevich (D-R), Temkin, Harkins-Jura, and Halsey isotherm models). It was found that the lead(II) adsorption on these samples followed the Freundlich, Dubinin-Radushkevich (D-R), and Halsey isotherm models. Moreover, the mean free energy of adsorption was calculated using Dubinin-Radushkevich (D-R) isotherm model and it was confirmed that the adsorption process was physical in nature. These findings are very meaningful in the removal of lead(II) ions from water using the hybrid membranes as adsorbents.

## 1. Introduction

Toxic heavy-metal ions, such as Cu^2+^, Pb^2+^, and Cd^2+^, dumped into water resources from industrial processes and household appliances, have caused a serious threat to the health of human body and organisms [1–3]. Water pollution caused by lead(II) ions from the spent liquor has especially caught increasing public attention. Consequently, the removal of lead(II) ions from water has turned out to be significantly important and highly urgent. To eliminate or reduce lead(II) pollution, various innovative approaches are designed and fabricated [2, 4–6]. Among these approaches, hybrid materials or polymers with functionalized groups disclose excellent adsorption affinities for heavy-metal ions when they are used as the hybrid adsorbents [5, 6]. However, the application of these new hybrids in the removal of heavy-metal ions from water still lacks sufficient theoretical principle. The relevant engineering data for their applications in industrial processes are especially deficient. Thus it will block their further applications in environmental aspects. These previous studies are therefore unsatisfactory. Further exploration from the theoretical model to application fundaments is of vital importance.

Recently, much effort was made to prepare new silica-based hybrids containing functionalized groups as adsorbents and characterize their adsorption properties for the toxic heavy-metal ions [7–9]. Some theoretical models and engineering data for their potential applications in industry are correlated and calculated in these previous jobs. Our continuing interest in these new silica-based hybrid adsorbents stimulates us to do more. Consequently, based on the previous study [9], herein, the isotherm models for lead(II) adsorption on silica-based hybrid membranes will be analyzed as typical toxic heavy-metal ions. The experimental data were correlated using six two-parameter isotherm equations (i.e., Langmuir, Freundlich, Dubinin-Radushkevich (D-R), Temkin, Harkins-Jura, and Halsey isotherm models) to probe the mechanism of lead(II) adsorption on hybrid membranes. We hope this investigation will be helpful in the effective disposal of heavy-metal ions from water using hybrid adsorbents.

## 2. Materials and Methods

### 2.1. Samples

3-Mercaptopropyl trimethoxysilane (MPTMS, purity: ≥95.0%) was purchased from Silicone New Material Co. Ltd. of Wuhan University (Wuhan, China) and used as received. Polyethylene glycol (PEG, MW: 10000) was purchased from National Pharmaceutical Group Corp. of China (Shanghai, China) and used as received. Acetaldehyde (40% wt in aqueous medium) and other reagents were of analytical grade.

### 2.2. Sample Preparation

**Scheme 1:**
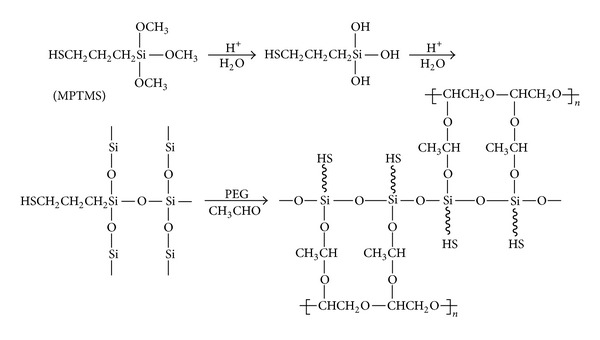
The possible preparation route of hybrid membranes used in this case [9].

The synthesizing procedure of sample A used in this case was described in detail in a previous article [9]. In this work, the samples A–D (the ratio of PEG (g) : MPTMS (mL) for samples A–D is 5.2 : 5, 5.2 : 10, 5.2 : 20, and 5.2 : 40, resp.) were used as adsorbents to investigate the isotherm models of lead(II) adsorption. For the convenience of study, the possible preparation route of these hybrid membranes is also given in Scheme 1.

### 2.3. Adsorption Experiment

The adsorption experiment was conducted in a way similar to the experiments described in detail in our previous articles, in which the lead(II) or copper(II) ions were adsorbed by the hybrid adsorbents [7–9]. The adsorption capacity (*q*_Pb^2+^_) of lead(II) ions on these adsorbents can be determined using

(1) $$\begin{array}{c} q_{\text{P}{\text{b}}^{\text{2+}}}=\frac{\left(C_{0}-C_{R}\right)V}{W}, \end{array}$$

where *V* (mL) is the volume of aqueous Pb(NO_3_)_2_ solution, *C*_0_ (mol dm^−3^) and *C*_*R*_ (mol dm^−3^) are the concentration of initial and remaining Pb(NO_3_)_2_, respectively, and *W* (g) is the weight of samples.

## 3. Results and Discussion

### 3.1. Adsorption Capacity versus Initial Concentration

**Figure 1:**
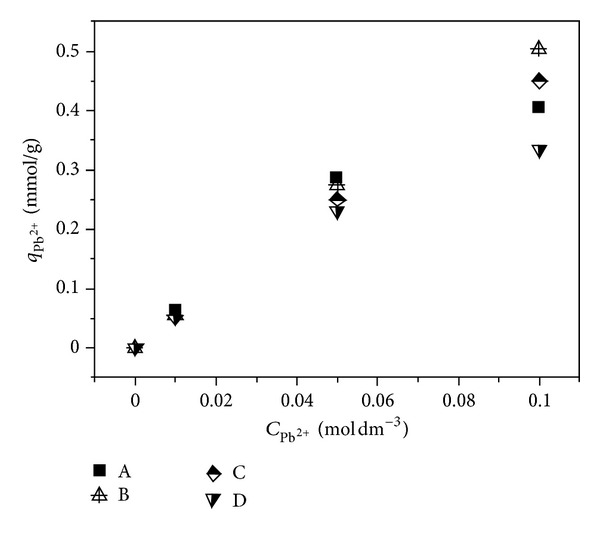
Lead(II) adsorption capacity versus initial concentration; the sample was immersed in different concentrations of aqueous Pb(NO_3_)_2_ solutions for 24 h, respectively.

The dependency of Pb^2+^ adsorption capacity on initial solution concentration was presented in Figure 1. 

As shown in Figure 1, it can be found that the adsorption capacity of lead(II) on samples A–D increases with an increase in the initial solution concentration. However, for the individual sample, the change trend in adsorption capacity is different. The reason can be assigned to adsorption property of samples.

To have an insight into the adsorption behaviors of lead(II) ions on samples A–D and to gain the optimal fitting of theoretical model, these experimental data were analyzed using six two-parameter isotherm equations (i.e., Langmuir, Freundlich, Dubinin-Radushkevich (D-R), Temkin, Harkins-Jura, and Halsey isotherm models), in which linear regression analysis was used to evaluate the better or the worse of the fitted theoretical model.

### 3.2. Langmuir Isotherm Model

Currently, it is well accepted that the Langmuir isotherm model is a typical monolayer adsorption, which can be applied to judge the adsorption properties of metal ions on the interface of a solid material. Generally, Langmuir isotherm equation can be expressed as (2) [5, 10], which is mainly based on the monolayer adsorption on the active reaction sites of the adsorbent:

(2) $$\begin{array}{c} \frac{C_{e}}{q_{e}}=\frac{C_{e}}{Q_{m}}+\frac{1}{Q_{m}b}, \end{array}$$

where *q*_*e*_ (mmol g^−1^) and *C*_*e*_ (mol dm^−3^) are the equilibrium concentrations of metal ions in the adsorbed and liquid phases, respectively. *Q*_*m*_ (mmol g^−1^) and *b* (dm^3^ mol^−1^) are the Langmuir constants, which can be calculated from the intercept and slope of the linear plot based on *C*_*e*_/*q*_*e*_ versus *C*_*e*_.

**Figure 2:**
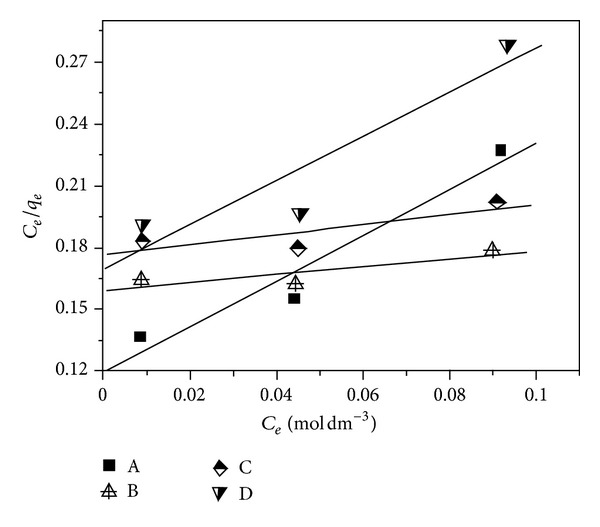
Plot of the Langmuir isotherm model for lead(II) adsorption on samples A–D.

Based on the relationship of adsorption capacity of lead(II) ions versus initial concentration illustrated in Figure 1, the Langmuir adsorption isotherms are modeled and presented in Figure 2. According to these isotherm curves, the Langmuir isotherm parameters are calculated and listed in Table 1. 

As shown in Table 1, it can be found that the values of linear regression coefficient (*R* ^2^) are located in the range of 0.669–0.941, suggesting that these experimental data fitted worse with the Langmuir isotherm equation. This finding indicates that lead(II) adsorption on samples A–D does not follow the Langmuir monolayer adsorption.

### 3.3. Freundlich Isotherm Model

Freundlich isotherm model is considered as the adsorption occurred on a heterogeneous surface with uniform energy, which can be expressed as [5, 10]

(3a) $$\begin{array}{c} q_{e}=k_{F}C_{e}^{1/n}, \end{array}$$

(3b) $$\begin{array}{c} \log\left(q_{e}\right)=\log k_{F}+\frac{1}{n}\log\left(C_{e}\right), \end{array}$$

where *q*_*e*_ (mmol g^−1^) and *C*_*e*_ (mol dm^−3^) are the equilibrium concentrations of metal ions in the adsorbed and liquid phases, respectively. *k*_*F*_ [(mmol g^−1^)(mol dm^−3^)^−1/*n*^] and *n* are the Freundlich isotherm constants, which can be calculated from the slope and intercept of the linear plot according to log⁡⁡(*q*_*e*_)  versus  log⁡⁡(*C*_*e*_).

**Figure 3:**
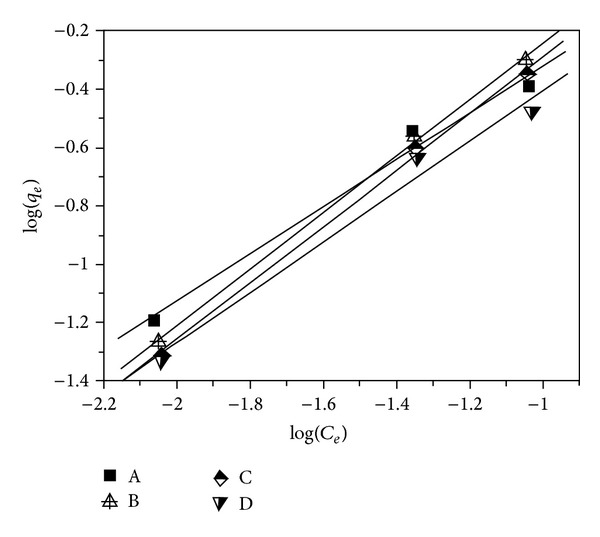
Plot of the Freundlich isotherm model for lead(II) adsorption on samples A–D.

Based on the relationship of adsorption capacity of lead(II) ions versus initial concentration (see Figure 1), the Freundlich adsorption isotherms are correlated and given in Figure 3. The Freundlich isotherm parameters are calculated and tabulated in Table 1. 

As shown in Table 1, it can be seen that the values of linear regression coefficient (*R* ^2^) are situated within the range of 0.983–0.998, demonstrating that the experimental data fitted well with the Freundlich isotherm equation. Moreover, it was reported [5, 10] that the Freundlich isotherm constant can be used to explore the favourability of adsorption process. When the value of n is within 1 < *n* < 10, it is favorable adsorption. If not, it is unfavorable adsorption. For the adsorption of lead(II) ions on samples A–D, it can also be seen in Table 1 that the values of n are situated in the range of 1–10, demonstrating that it is favorable for the adsorption process.

These outcomes evidence that the lead(II) adsorption on the samples A–D followed the Freundlich isotherm model and such adsorption mainly occurred on the heterogeneous surface of the samples.

### 3.4. Dubinin-Radushkevich (D-R) Isotherm Model

To predict the adsorption effect, that is, physical or chemical adsorption, the mean free energy of adsorption was calculated by the Dubinin-Radushkevich (D-R) isotherm equation, which can be expressed as [11, 12]

(4a) $$\begin{array}{c} q_{e}=Q_{\text{DR}}\exp\left(-K_{\text{DR}}{\left[RT\ln\left(1+\frac{1}{C_{e}}\right)\right]}^{2}\right), \end{array}$$

(4b) $$\begin{array}{c} \ln\left(q_{e}\right)=\ln Q_{\text{DR}}-K_{\text{DR}}{\left[RT\ln\left(1+\frac{1}{C_{e}}\right)\right]}^{2}, \end{array}$$

where *q*_*e*_ (mmol g^−1^) is the amount of metal ions adsorbed, *Q*_DR_ (mmol g^−1^) is the maximum adsorption capacity of metal ions, *K*_DR_ (mol^2^/kJ^2^) is the Dubinin-Radushkevich isotherm constant, *C*_*e*_ (mol dm^−3^) is the equilibrium concentration of metal ions, *R* is the gas constant (8.314 J/mol K), and *T* (K) is absolute temperature in Kelvin.

The Dubinin-Radushkevich (D-R) isotherm constant, *K*_DR_, is related to the mean free energy of adsorption, *E* (kJ/mol), which can be obtained using the following expression [11]:

(5) $$\begin{array}{c} E=\frac{1}{\sqrt{2K_{\text{DR}}}}. \end{array}$$

**Figure 4:**
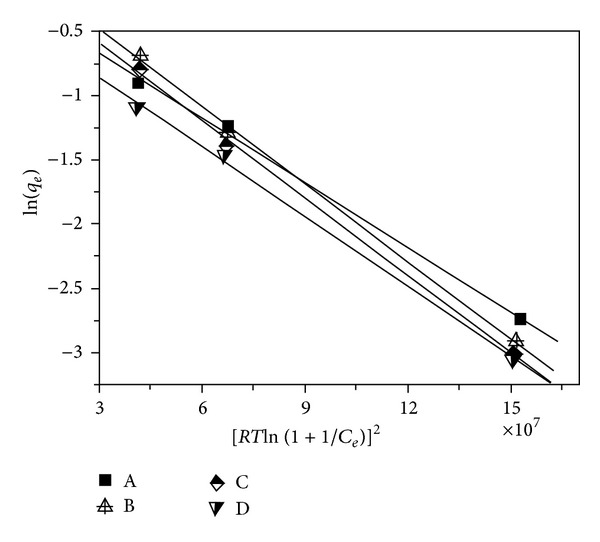
Plot of the D-R isotherm model for lead(II) adsorption on samples A–D.

Making the linear plot according to lead(II) adsorption capacity versus initial concentration (as illustrated in Figure 1), the Dubinin-Radushkevich (D-R) adsorption isotherms can be obtained and are presented in Figure 4. Corresponding to these linear plots, the Dubinin-Radushkevich (D-R) isotherm parameters are calculated and summarized in Table 1.

As summarized in Table 1, it can be observed that the values of linear regression coefficient (*R* ^2^) are in the range of 0.997–0.998, revealing that the experimental data fitted well with the Dubinin-Radushkevich (D-R) isotherm model. Moreover, it is reported in some articles [3, 11–13] that, when the value of *E* is below 8 kJ/mol, the adsorption process can be considered as the physical adsorption. In contrast, if the value of *E* is located in the range of 8–16 kJ/mol, it is the chemical adsorption. From Table 1, it can be observed that the obtained values of mean free energy, *E*, are limited within the range of 4.98–5.45 kJ/mol. Based on these data, it can thus be concluded that the effect of physical adsorption will play a dominating role in the adsorption process of lead(II) ions on samples A–D.

### 3.5. Temkin Isotherm Model

Temkin isotherm model is a useful tool to estimate the adsorption heat, which can be calculated using the following equation [13, 14]:

(6) $$\begin{array}{c} q_{e}=\frac{RT}{b_{T}}\ln\left(K_{T}C_{e}\right)=B_{T}\ln\left(K_{T}C_{e}\right), \end{array}$$

where constant *B*_*T*_ = *RT*/*b*_*T*_, which is related to the adsorption heat, *R* is the gas constant (8.314 J/mol K), *T* (K) is absolute temperature in Kelvin, *b*_*T*_ (J/mol) is the Temkin isotherm constant, which is the variation of adsorption energy and *K*_*T*_ is the equilibrium binding constant corresponding to the maximum binding energy. Both *B*_*T*_ and *K*_*T*_ can be calculated from the slope and the intercept of the linear plot based on *q*_*e*_  versus  ln⁡(*C*_*e*_), respectively.

**Figure 5:**
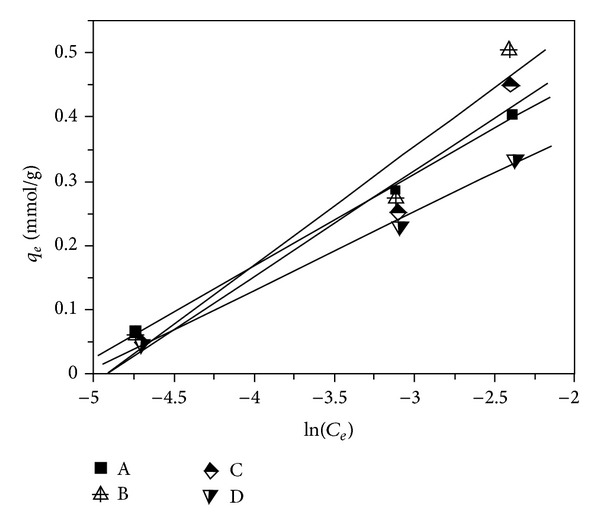
Plot of the Temkin isotherm model for lead(II) adsorption on samples A–D.

Figure 5 illustrates the Temkin isotherm model for lead(II) adsorption on samples A–D. The relevant isotherm parameters are listed in Table 1. 

It can be discovered in Table 1 that the values of *R* ^2^ are positioned within 0.946–0.998, which gave a close fit to the lead(II) adsorption on samples A–D. This outcome suggests that the experimental data fitted better with the Temkin isotherm model. Furthermore, it can also be discovered in Table 1 that the adsorption heat of lead(II) adsorption on samples A–D was restricted within 14–22 kJ/mol.

### 3.6. Harkins-Jura Isotherm Model

It is reported [3, 13] that Harkins-Jura isotherm model mainly describes the multilayer adsorption and the existence of the heterogeneous pore distribution in the surface of adsorbents, which can be expressed as

(7) $$\begin{array}{c} \left[\frac{1}{q_{e}^{2}}\right]=\left[\frac{B_{\text{HJ}}}{A_{\text{HJ}}}\right]-\left[\frac{1}{A_{\text{HJ}}}\right]\log\left(C_{e}\right), \end{array}$$

where *B*_HJ_ and *A*_HJ_ are the Harkins-Jura constants. Both *B*_HJ_ and *A*_HJ_ can be achieved from the slope and the intercept of the linear plot based on 1/*q*_*e*_^2^  versus  log⁡(*C*_*e*_), respectively.

**Figure 6:**
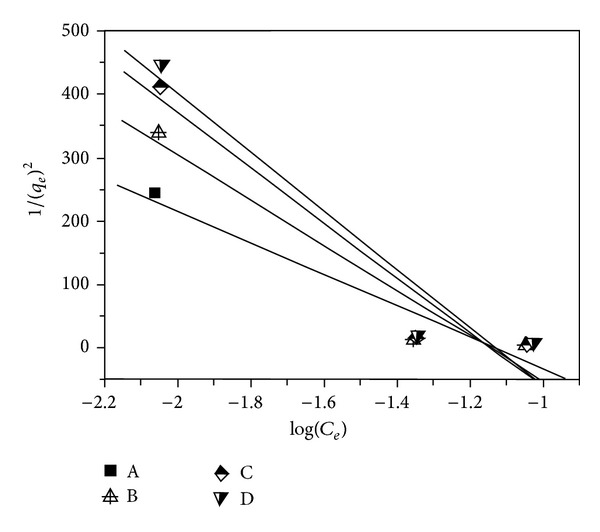
Plot of the Harkins-Jura isotherm model for lead(II) adsorption on samples A–D.

The Harkins-Jura isotherm model for lead(II) adsorption on samples A–D is presented in Figure 6 and the relevant isotherm parameters are calculated and summarized in Table 1.

It can be noted in Table 1 that the values of *R* ^2^ are located in the range of 0.920–0.925, which indicate the worse fits to the lead(II) adsorption on samples A–D. This result reveals that lead(II) adsorption on samples A–D is against the rule of multilayer adsorption.

### 3.7. Halsey Isotherm Model

Halsey isotherm model can be used to evaluate the multilayer adsorption system for metal ions adsorption at a relatively large distance from the surface [3, 13], which can be calculated using

(8) $$\begin{array}{c} \ln\left(q_{e}\right)=\left[\left(\frac{1}{n_{H}}\right)\ln\left(K_{H}\right)\right]-\left(\frac{1}{n_{H}}\right)\ln\left(\frac{1}{C_{e}}\right), \end{array}$$

where *K*_*H*_ and *n*_*H*_ are the Halsey constants, which can be obtained from the slope and the intercept of the linear plot based on ln⁡(*q*_*e*_)  versus  ln⁡(*C*_*e*_), respectively.

**Figure 7:**
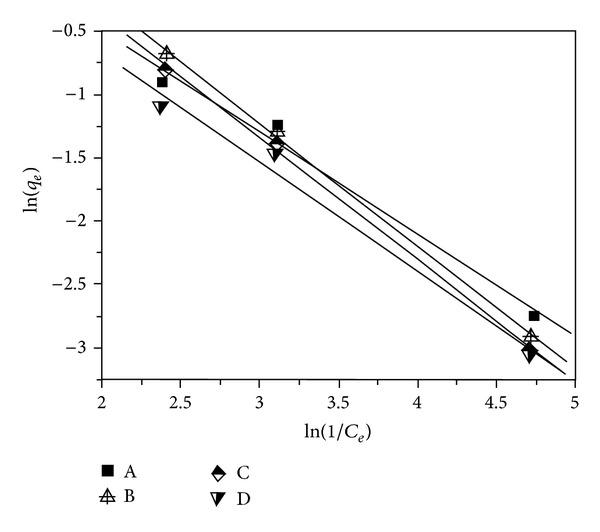
Plot of the Halsey isotherm model for lead(II) adsorption on samples A–D.

Figure 7 gives the Halsey isotherm model for lead(II) adsorption on samples A–D. The related Halsey isotherm parameters are calculated and tabulated in Table 1.

It can be detected in Table 1 that the values of *R* ^2^ are centered in the region of 0.983–998, which show the best fits to the lead(II) adsorption on samples A–D. This finding implies that lead(II) adsorption on samples A–D obeys the Halsey isotherm model.

Notice that, by comparing the values of linear regression coefficient (*R* ^2^) of the examined six isotherm models, it can be concluded that the Freundlich, Dubinin-Radushkevich (D-R), and Halsey isotherm models gave much better fitting than the other three isotherm models. Consequently, the adsorption behaviors of lead(II) ions on samples A–D can be well described using these three isotherm models.

## 4. Conclusions

The lead(II) adsorption isotherms are modeled using six two-parameter isotherm equations. The following results can be achieved.

1. The adsorption of lead(II) ions on these samples followed the Freundlich, Dubinin-Radushkevich (D-R), and Halsey isotherm models.
2. The mean free energy of adsorption, *E*, calculated from Dubinin-Radushkevich (D-R) isotherm equation, was in the range of 4.98–5.45 kJ/mol. This result suggested that it is physical adsorption process.
3. The adsorption heat calculated from the Temkin isotherm equation was restricted within 14–22 kJ/mol.

## Figures and Tables

**Table 1:** The Langmuir, Freundlich, Dubinin-Radushkevich (D-R), Temkin, Harkins-Jura, and Halsey isotherm parameters for lead(II) adsorption.

Parameter	Values			
	Sample A	Sample B	Sample C	Sample D
Langmuir isotherm model				
*Q*_*m*_ (mmol/g)	0.898	5.335	4.106	0.935
*b *	9.358	1.175	1.379	6.303
*R* ^2^	0.941	0.722	0.669	0.854

Freundlich isotherm model				
*k*_*F*_	3.061	5.380	4.754	2.875
*n *	1.240	1.030	1.034	1.158
*R* ^2^	0.983	0.998	0.998	0.984

Dubinin-Radushkevich (D-R) isotherm model				
*Q*_DR_ (mmol g^−1^)	0.846	1.124	1.007	0.733
*K*_DR_ (mol^2^/kJ^2^)	0.0168	0.020	0.020	0.0181
*E* (kJ/mol)	5.455	4.990	4.989	5.252
*R* ^2^	0.997	0.998	0.998	0.997

Temkin isotherm model				
*b*_*T*_ (kJ/mol)	18.154	14.125	15.768	21.385
*B*_*T*_	0.143	0.184	0.165	0.121
*K*_*T*_	175.878	136.724	136.521	158.936
*R* ^2^	0.998	0.946	0.953	0.996

Harkins-Jura isotherm model				
*A*_HJ_	0.00402	0.00280	0.00230	0.00216
*B*_HJ_	−1.131	−1.149	−1.144	−1.130
*R* ^2^	0.920	0.925	0.924	0.920

Halsey isotherm model				
*K*_*H*_	4.005	5.659	5.013	3.399
*n*_*H*_	1.240	1.030	1.034	1.158
*R* ^2^	0.983	0.998	0.998	0.984
